# Updated MDSGene review on the clinical and genetic spectrum of *LRRK2* variants in Parkinson´s disease

**DOI:** 10.1038/s41531-025-00881-9

**Published:** 2025-02-17

**Authors:** Clara Krüger, Shen-Yang Lim, Alissa Buhrmann, Fenja L. Fahrig, Carolin Gabbert, Natascha Bahr, Harutyun Madoev, Connie Marras, Christine Klein, Katja Lohmann

**Affiliations:** 1 Institute of Neurogenetics, University of Lübeck, Lübeck, Germany; 2 Division of Neurology, Department of Medicine, Faculty of Medicine, University of Malaya, Kuala Lumpur, Malaysia; 3 The Mah Pooi Soo & Tan Chin Nam Centre for Parkinson’s & Related Disorders, University of Malaya, Kuala Lumpur, Malaysia; 4 Edmond J Safra Program in Parkinson’s Disease, University Health Network, University of Toronto, Toronto, Canada

**Keywords:** Medical genetics, Parkinson's disease

## Abstract

Pathogenic variants in the *LRRK2* gene are one of the most commonly identifiable monogenic causes of Parkinson´s disease (PD, PARK-LRRK2). This systematic MDSGene literature review comprehensively summarizes published demographic, clinical, and genetic findings related to *LRRK2* variants (https://www.mdsgene.org/). Data on 4660 individuals with 283 different variants were curated. The median age at onset in the PD patients with available information was 56 years, notably, with approximately one-third having PD onset <50 years. Tremor was the most frequently reported initial symptom and more common than reported in other dominantly inherited forms of PD. Of the 211 potentially PD-causing variants, 25 were classified as pathogenic or likely pathogenic, and the remaining 186 (88.2%) were variants of uncertain significance. p.G2019S was the most frequently reported pathogenic variant, followed by p.R1441G and p.R1441C. This systematic review represents the most extensive database on PARK-LRRK2 to date and provides a vital resource to improve precision medicine.

## Introduction

Parkinson’s disease (PD) is a relatively common, age-related, neurodegenerative disorder characterized by motor (bradykinesia, resting tremor, rigidity, and postural instability) and non-motor features (neuropsychiatric features, autonomic symptoms, sleep disorders, and sensory dysfunction)^1^. The age at onset for almost 25% of affected individuals is younger than 65 years, and for 5–10%, younger than 50 years^2^. Causes of PD include genetic and environmental factors as well as interactions thereof^2^. Monogenic causes, i.e., pathogenic variants in genes such as *LRRK2, SNCA, VPS35, PRKN, PINK1*, and *PARK7*, often play a role in patients with early-onset disease^3,4^. Further, coding risk variants and rare pathogenic variants (often with highly reduced penetrance)^5^ in the *GBA1* gene are found in about 4–20% of PD patients^6^. Although monogenic forms comprise a minority of all PD patients^7–9^, they are important as affected individuals are the most likely candidates for potential gene-specific, targeted treatments. These treatments are currently being evaluated in trials^2^, including kinase inhibitors for PD linked to pathogenic variants in the *LRRK2* gene (PARK-LRRK2)^10,11^.

Pathogenic variants in the *LRRK2* gene are one of the most frequent causes of dominantly inherited PD^12^. *LRRK2* (Leucine-Rich Repeat Kinase 2) encodes a multidomain protein kinase that shows autophosphorylation^13^. It plays an important role in different cellular processes, such as cytoskeleton remodeling, vesicular trafficking, autophagy, and protein translation^14^. Increased LRRK2 kinase activity is thought to dysregulate these processes resulting in the death of dopaminergic neurons in the *substantia nigra*^15^. Until recently, only seven variants in *LRRK2* were considered clearly pathogenic (p.N1437H, p.R1441C/G/H, p.Y1699C, p.G2019S, and p.I2020T)^4,16,17^. These variants are mainly located in the guanine triphosphatase (GTPase) Ras-of-complex (ROC) and the kinase domain, which represent two functionally linked enzymatic domains^12,18^. More recently, in-vivo and in-vitro functional testing of LRRK2´s kinase activity became available and revealed ~20 additional variants that lead to increased LRRK2 kinase activity and are thus plausible contributors to the pathogenesis of PD^18,19^. Importantly, the increased LRRK2 kinase activity represents a possible therapeutic target^10,20^. In addition to these pathogenic kinase-activating, gain-of-function variants in *LRRK2*, there are also loss-of-function variants, i.e., nonsense and frameshift variants. However, these truncating variants in *LRRK2* are found with similar frequency in PD patients and controls, indicating that *LRRK2* loss-of-function variants may not alter the risk for PD^21,22^. Further, copy number variants (CNVs), especially larger deletions, are extremely rarely found in *LRRK2*^23,24^.

The very large number of scientific publications related to PARK-LRRK2 (>3000), makes it overwhelming and challenging to follow the literature. Despite the large body of literature, there remain many uncertainties, especially with respect to the phenotypic spectrum and the interpretation of the many genetic variants. These knowledge gaps potentially hamper the identification of PARK-LRRK2 patients who would be eligible for gene-specific clinical trials. Here, we provide a systematic literature review using the protocol of the Movement Disorders Society Genetic Mutation Database (MDSGene) (https://www.mdsgene.org/)^25^. With this review, we update and considerably extend the MDSGene database to over 200 potentially disease-causing *LRRK2* variants reported in more than 3000 PD patients. The numbers of included variants and patients have increased approximately tenfold and fourfold, respectively, compared to the initial review covering publications until 2017^4^.

## Results

### Included articles, study types, and mutation screening methods

**Fig. 1:**
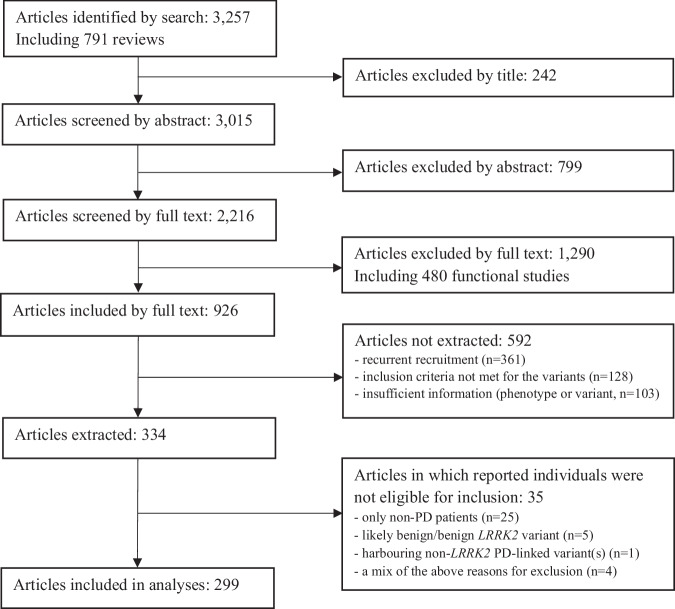
Flowchart of the literature search. The number of included and excluded articles at the different steps is indicated.

We identified 3257 publications through the search strategy, carried out for the last time in PubMed on October 15th, 2024, and supplemented with articles from HGMD professional v2022.4. An overview of the literature search and the filtering process is shown in Fig. 1. At screening, 926 articles were considered eligible for data abstraction. However, 592 did not contain relevant information (Fig. 1). The full text of 334 papers was reviewed, and 299 of these publications included patients who fulfilled the inclusion criteria (Fig. 1).

The most frequently included study types were mutational screens (*n* = 168, 56.2%), followed by case reports or case series (*n* = 46, 15.4%), association studies (*n* = 36, 12.0%), family studies (*n* = 16, 5.4%), and a combination of different designs (*n* = 33, 11.0%).

**Fig. 2:**
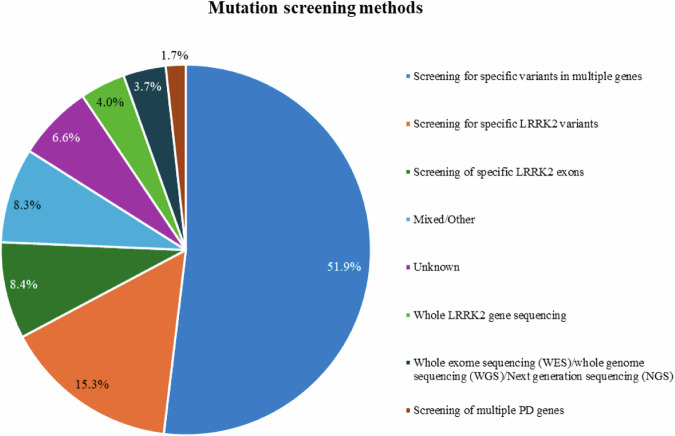
Mutation screening methods. Frequencies of the mutation screening methods in the included patients.

A variety of genetic screening methods have been applied to detect *LRRK2* variants, ranging from screening for selected *LRRK2* variants only (15.3%) to whole exome or genome sequencing (3.7%, Fig. 2). Most studies (51.3%) screened for selected variants in different PD genes (51.9%).

### Pathogenicity scoring

**Fig. 3:**
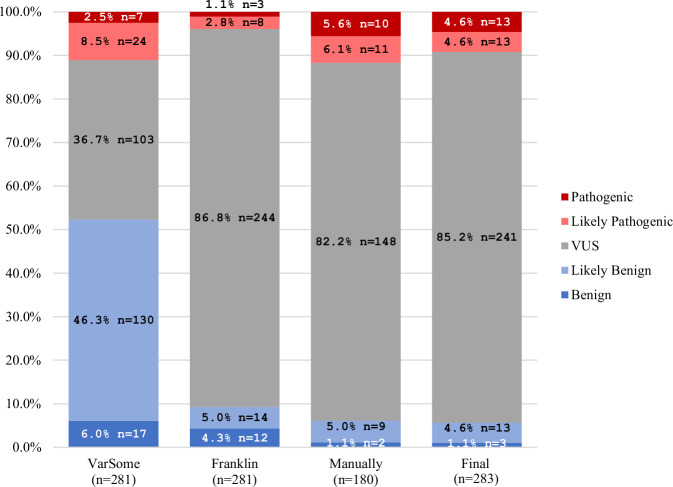
Results of the pathogenicity scoring. Comparison of the ACMG-based pathogenicity scoring of all *LRRK2* variants from VarSome, Franklin, manual ACMG pathogenicity scoring, and the final pathogenicity scoring.

A total of 283 *LRRK2* variants were identified, of which 281 were scorable using VarSome and Franklin. The remaining two variants and 178 of the other variants were manually scored following the ACMG recommendations^26^. There were considerable discrepancies in the pathogenicity scoring between VarSome and Franklin (Fig. 3). The manually scoring was closer to Franklin (Fig. 3). One reason for the discrepancies was an underestimation in VarSome, which interpreted many VUS erroneously as “likely benign” since the criterion BP1 was applied (“Missense variant in a gene for which primarily truncating variants are known to cause disease”). This is not applicable to *LRRK2*, since loss of function of the protein is not a known disease mechanism^21,22^. In fact, many missense variants in *LRRK2* have instead been demonstrated to be functionally relevant variants^18^. Using BP1 resulted in scoring almost half of the variants as likely benign by VarSome. Another discrepancy can be explained by an underestimation of likely pathogenic variants, being classified as VUS, in Franklin since the criterion PS3 was not applied (“Well-established in-vitro or in-vivo functional studies supportive of a damaging effect on the gene or gene product.”), even though functional evidence for these variants has become available^18^. In the combined and finally considered classification, the vast majority of the variants were classified as VUS (241/283, 85.2%; Fig. 3). A list of all *LRRK2* variants can be found in Supplementary Table 1.

### Included patients and variants

**Fig. 4:**
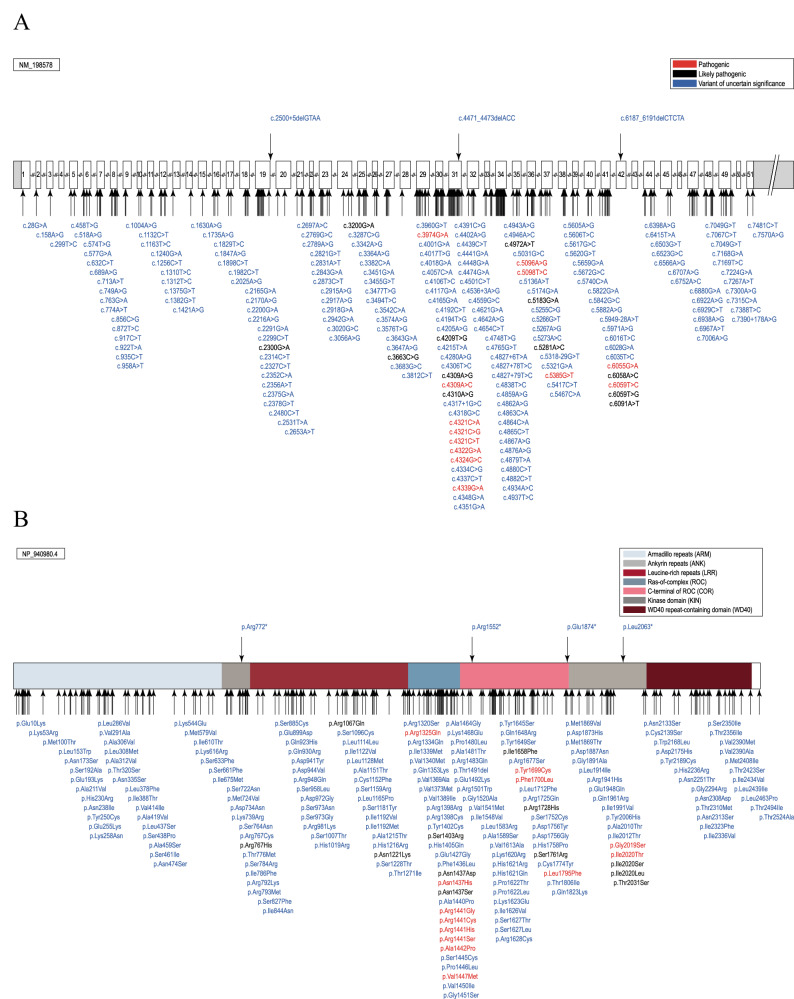
Potentially pathogenic variants in *LRRK2.* Schematic representation of the *LRRK2* gene (**A**) and protein (**B**) with the localization of the included variants. The position of the variants is indicated by arrows, and the predicted pathogenicity is provided by color (red, pathogenic; black, likely pathogenic; blue, variants of uncertain significance (VUS). Protein domains in B are color-coded.

A total of 4660 *LRRK2* variant carriers were identified, and 3387 PD patients with a potentially pathogenic *LRRK2* variant (72.7%) were included in MDSGene and the analyses, based on the inclusion and exclusion criteria. Among the 4660 individuals, 76.3% (*n* = 3555) of them had PD, 1.4% (*n* = 66) had other diseases (see below), and 22.3% (*n* = 1039) were clinically unaffected. The included 3387 PD patients carried 211 different variants, of which 13 have been classified as pathogenic (6.2%), 12 as likely pathogenic (5.7%), and 186 as VUS (88.2%; Fig. 4).

Of the 211 included variants, missense variants (*n* = 194, 91.9%) were the most frequent variant type, followed by intronic variants (*n* = 5, 2.4%), nonsense variants (*n* = 4, 1.9%), splice region variants (*n* = 4, 1.9%), silent changes (*n* = 2, 0.9%), splice site variants (*n* = 1, 0.5%), and small deletions (*n* = 1, 0.5%). Of note, all variants other than missense variants were classified as VUS. The most frequent variant among the included patients was the p.G2019S substitution, classified as pathogenic, that was present in 2480 patients (73.2%). Notably, among the clinically unaffected individuals, 743 (71.5%) carried this missense variant, i.e., 743 of 3223 (23.1%) p.G2019S carriers were unaffected (age at examination unknown for 644 of the unaffected individuals, 86.7%). The second most frequently reported variant among the included patients was p.R1441G in 195 patients (5.8%). This variant was also reported in 90 unaffected individuals, which means that 90 of the 285 (31.6%) reported carriers were unaffected (Supplementary Table 2). Since unaffected variant carriers are not systematically reported and have not been extracted from the beginning of the project^4^, no definite conclusion on the penetrance of these variants can be drawn. Nevertheless, incomplete penetrance for p.G2019S and also for p.R1441G plays a considerable role since a large proportion of individuals with these variants had not developed manifest PD at the point of inclusion.

### Excluded patients and variants

Notably, among the 4660 individuals with *LRRK2* variants, 0.4% (*n* = 18) had an atypical parkinsonian disorder, and 1.0% (*n* = 48) had other diseases (such as immunological diseases, cancer, dementia, or essential tremor). These were excluded from the analyses since the sole causative role of the *LRRK2* genotype in these non-PD phenotypes has not been established.

Among the 66 patients with a non-PD phenotype extracted during the systematic literature review, 8 patients also carried a potentially disease-causing variant in another gene. Of the remaining 58 patients, only 20 carried pathogenic or likely pathogenic variants, mostly p.G2019S, 36 carried VUS, and 2 benign or likely benign variants. The phenotypic spectrum of carriers with (likely) pathogenic variants was broad, including MSA (*n* = 3)^27,28^, PSP (*n* = 3)^29–31^, CBD (*n* = 1)^32^, dystonia (*n* = 2)^33,34^, Alzheimer´s disease (*n* = 1)^35^, amyotrophic lateral sclerosis (*n* = 2)^36^, restless legs syndrome (*n* = 1)^37^, schizophrenia (*n* = 1)^38^, multiple sclerosis (*n* = 3)^39^, rheumatoid arthritis (*n* = 1)^39^, achalasia (*n* = 1)^39^, and breast cancer (*n* = 1)^40^. Often, these non-PD phenotypes were found in relatives of PD patients with the same *LRRK2* variant. Of note, some of these phenotypes are frequent. Thus, it is also conceivable that they co-occur by chance, and these patients show reduced penetrance of the *LRRK2* variant in terms of PD.

Regarding the 18 patients with atypical parkinsonian disorders, 7 patients carried pathogenic *LRRK2* variants (see above), 8 carried VUS, and 3 had benign or likely benign *LRRK2* variants. Among the 15 carriers with potentially disease-causing *LRRK2* variants were 7 PSP, 5 MSA, 2 CBD, and 1 DLB patient(s).

We further excluded 46 patients whose genetic cause could not be unequivocally assigned to *LRRK2* since they carried potentially disease-related variants in at least one other PD gene. These variants could have an additive effect on the phenotypic presentation, potentially acting as a component of oligogenic causes^41^. The excluded patients comprised 20 patients with an additional, pathogenic variant in *GBA1* (p.N409S or p.L483P), and 12 patients with an additional heterozygous or homozygous *PRKN* variant. Notably, one patient, who, to our knowledge, is the only case in the literature carrying a large deletion in *LRRK2* (homozygous Exon 49 deletion), also carried a homozygous, pathogenic deletion of Exon 4 in *PRKN*^24^. A list of the excluded PD patients with *LRRK2* and additional potentially pathogenic variants in other PD genes can be found in Supplementary Table 3.

### Demographic and phenotypic Data

The phenotypic and demographic data in the next paragraph are based on observations in carriers of pathogenic or likely pathogenic variants only (*n* = 2981), unless not otherwise stated. The corresponding information on carriers of VUS (*n* = 406) and all included patients (*n* = 3387) can be found in the Supplementary Material.

#### Demographic data

Information on age at examination was available for 695 patients (23.3%) who had a median age of 67 (range 34–95, interquartile range: 58–75) years (the mean age with standard deviation was 66.1 ± 12.1 years). Based on 1340 included patients with information, 51.3% (*n* = 688) were male. Patients were most commonly European/White (34.1%), Arab (26.8%), or of mixed/other ethnicities (15.4%), based on 1479 patients with available information (Supplementary Fig. 1). Most reported patients originated from Tunisia (30.1%), Spain (17.1%), Italy (7.3%), or the USA (5.2%) (Supplementary Fig. 1). Notably, among the Tunisian patients, all (545/545, 100.0%) carried the p.G2019S variant (median AAO 58 years), while in Spain, about half of the patients carried this variant (148/310, 47.7%, median AAO 62 years) and the other half the p.R1441G variant (155/310, 50.0%, median AAO 56 years). For country-specific distribution of variants, see https://www.mdsgene.org/d/41/g/4?action=plot_map&fc=0&_mu=1&_country=1. A positive family history was reported for almost a third (32.5%) of the PD patients with (likely) pathogenic *LRRK2* variants, it was negative for 19.5% and unknown or not reported for 48.0%.

#### Age at onset

**Fig. 5:**
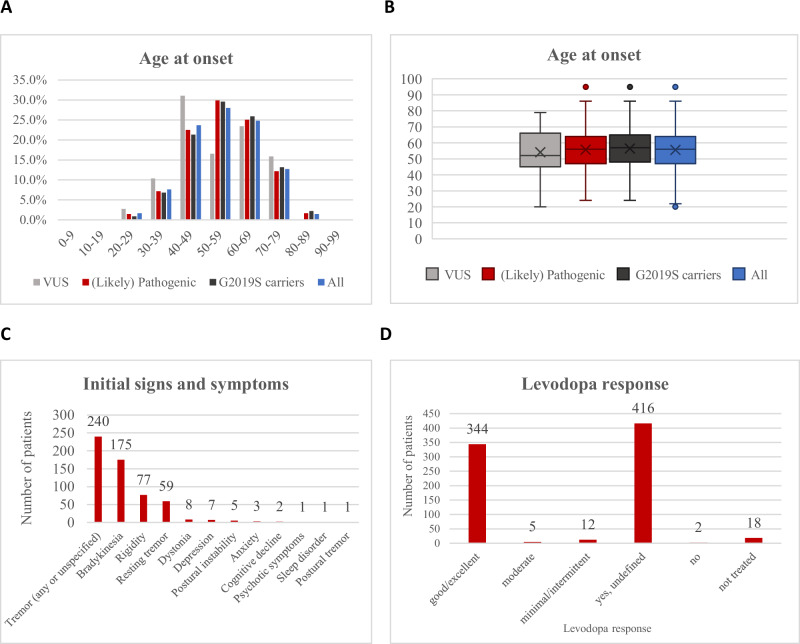
Other clinical features in the included patients. **A** The age at onset (AAO) distribution is shown in 10-year bands on the x-axis. The percentage of patients is displayed on the y-axis. The graph shows the distribution for all included PD patients (blue), for the patients with VUS only (gray), for the patients with the p.G2019S variant (black), and for patients with (likely) pathogenic variants (red). **B** Box plots for the AAO in years in the four groups, depicting medians and interquartile ranges. Outlier points are also displayed and are defined as data points that lie outside 1.5 times the interquartile range (IQR) from the lower or upper quartile boundary. **C** Initial signs and symptoms in the included patients with pathogenic or likely pathogenic variants. **D** Levodopa response quantifications in the included PD patients with pathogenic or likely pathogenic variants. The x-axis shows the six divisions of the levodopa response quantifications and the y-axis the number of patients.

Age at onset (AAO) was available for 894 patients (30.0%). The median age at onset (AAO) was 56 (range 24–95, interquartile range: 47–64) years (Fig. 5A). While the majority of patients (68.9%; *n* = 616) had a late onset (≥50 years), approximately one-third (31.1%; *n* = 278) had early-onset PD (EOPD), defined as an AAO after 21 and before 50 years of age^42^. Notably, 8.6% (*n* = 77) of patients were reported with an AAO < 40 years. Although one patient was reported to have an AAO of 10 years^43^, it was actually 25 years (personal communication of Dr Lanza). The median disease duration was 10 (range 0–42) years. When analyzing only carriers of VUS, the median AAO was 52 (range 20–79) years, suggesting that at least a portion of the VUS might also act as a driver of the disease since this AAO is younger than that reported for PD in general^44^. The median AAO of p.G2019S carriers was 57 (range 24–95) years (Fig. 5B).

#### Signs and symptoms

**Fig. 6:**
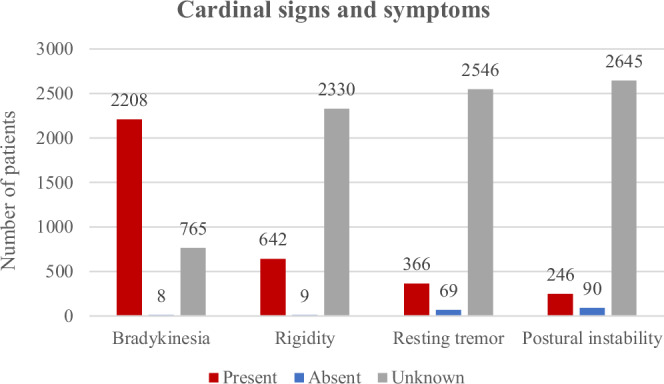
Signs and symptoms in the included patients at last examination. Cardinal clinical signs and symptoms in the included PARK-LRRK2 patients with pathogenic or likely pathogenic variants.

The data extraction covered 14 motor and seven non-motor symptoms (Supplementary Table 5). Motor symptoms were present in all included patients (per inclusion criterion). In contrast, at least one non-motor symptom was reported in 9.3%, indicated as absent in 0.9%, and information was missing for 89.7% of the patients. The most frequently reported motor symptom was bradykinesia, which was indicated as present in 74.1% of the patients and absent in 0.3% (according to the authors, who nevertheless established a diagnosis of PD). This was followed by rigidity (present in 21.5%, absent in 0.3%) and resting tremor (present in 12.3%, absent in 2.3%). Postural instability was the least reported of the four cardinal clinical features and was reported to be present in only 8.3% of the patients (Fig. 6). Among the non-cardinal motor symptoms, dyskinesia (present in 6.9%; absent in 3.1%; median disease duration in patients with dyskinesias: 14 years), motor fluctuations (present in 4.9%; absent in 2.4%; median disease duration in patients with motor fluctuations: 13.5 years), and dystonia (present in 2.2%; absent in 3.4%) were still relatively frequently reported, but the others were only reported to be present in less than 1% of the included patients. Among the non-motor symptoms, cognitive decline and depression were the most frequently reported, each present in 3.7% of the patients (median disease duration 11 years (cognitive decline) and 10 years (depression) (Supplementary Table 6). There were no clear differences regarding the frequency of motor and non-motor features in relation to the interpretation of the variant, i.e., (likely) pathogenic (83.2% of this group were carriers of p.G2019S) vs. VUS (Supplementary Tables 6, 7, Supplementary Fig. 2).

Initial signs and symptoms were reported for 15.3% of the patients only. The most common initial symptom was tremor, which was present in 52.5% of these patients (Fig. 5C). This was followed by bradykinesia (38.3%), and rigidity (16.8%). No clear differences were observed between carriers of variants considered (likely) pathogenic or VUS (Supplementary Table 9, Supplementary Fig. 3). Further subgroup analyses, for instance, for individual variants, were not meaningful due to the small number of carriers and high degree of missing data.

All demographic, clinical, and genetic information is available on the MDSGene website (https://www.mdsgene.org/) and can also be filtered by specific variants and their different classifications of pathogenicity.

#### Comparison to other dominantly inherited forms of PD

When comparing features of PARK-LRRK2 to other dominantly inherited forms of PD, i.e., PARK-SNCA or PARK-VPS35, it was noticeable that for all analyzed clinical features, *SNCA* and *VPS35* variant carriers had a higher proportion of symptoms reported as present than *LRRK2* variant carriers (https://www.mdsgene.org/). In terms of initial presentation, the only difference found between PARK-LRRK2, PARK-SNCA, and PARK-VPS35 was that the most common sign and symptom in both PARK-SNCA (31.2%) and PARK-VPS35 patients (15.9%) was bradykinesia, while in *LRRK2* variant carriers, it was tremor (52.5%). Apart from tremor as an initial symptom in PARK-LRRK2, the identification of other clinical clues to the possible presence of PARK-LRRK2 was hampered by the high proportion of missing information in the published literature and needs to be an area of ongoing and future work.

#### Levodopa treatment response

For 26.7% of the patients (*n* = 797), information was available on whether levodopa treatment was administered (yes for *n* = 777). No information on the treatment response was provided for about half of them (53.5%). For those patients with information (*n* = 361), almost all (95.3%) had a good/excellent response to levodopa (Fig. 5D), with no apparent difference to carriers of VUS (Supplementary Table 10, Supplementary Fig. 4). Information on other therapies, such as pump therapy or surgical treatment, including deep brain stimulation (DBS), was rarely available^45^ and not systematically analyzed. Reports by other investigators^46,47^, including pooled analyses^48,49^, indicate a beneficial effect of DBS in PARK-LRRK2.

## Discussion

This systematic literature review provides a comprehensive and up-to-date review of demographic, clinical, and genetic findings from the published literature on PARK-LRRK2. It is based on curated data from 3387 PD patients with 211 different potentially disease-causing variants in *LRRK2*. This represents, by far, the largest database on PARK-LRRK2 to date. A comprehensive overview of *LRRK2* variants and interpretation is highly relevant to clinicians in establishing a possible diagnosis of PARK-LRRK2. This becomes increasingly actionable in light of the identification of several LRRK2 kinase inhibitors (MLi-2 (Merck), DNL-201, DNL-151/BIIB122 (Genentech/Denali), and PF-360 (Pfizer)), including first clinical trials for DNL-201 and DNL-151/BIIB122. The frequent discrepancies between variant interpretation using two automated online tools (i.e., Franklin and Varsome) highlight the value of an expert panel-guided variant interpretation for clinicians and genetic counselors.

Such a comprehensive review can help to identify clinical features to differentiate PARK-LRRK2 from idiopathic PD and other monogenic forms. Notably, the median AAO for PARK-LRRK2 in this systematic review was 56 years, lower than the median AAO of 60–75 years reported for PD patients in general, consistent with the notion that monogenic causes for PD typically have earlier AAO^50^. Remarkably, around one-third of patients had EOPD with onset below age 50 years, thus adding nuance to a prevailing view that *LRRK2* variants cause late-onset PD^51,52^. Compared to other genes linked to autosomal dominantly inherited PD, PARK-LRRK2 has a later median AAO vs. PARK-SNCA (46 years) but a similar AAO to PARK-VPS35 (52 years) patients. Genetic forms linked to autosomal recessive PD (*PARK7, PINK1, PRKN*) show even earlier median AAO (27, 31, 32 years; https.//www.mdsgene.org). Further, the reported prevalences of motor response complications and cognitive impairment in PARK-LRRK2 appeared lower in comparison to idiopathic PD. Dyskinesias and motor fluctuations were present in 6.9% and 4.9% of PARK-LRRK2 patients, respectively (with median disease duration of 14 and 13.5 years), relatively low compared with the widely quoted estimate of ~10% of levodopa-treated PD patients per year developing motor response complications^53–55^. Similarly, although cognitive decline was the most frequently reported non-motor symptom in this systematic review, it was reportedly present in only 3.7% of patients (median disease duration 11 years), considerably lower than most published studies of idiopathic PD (for example, a recent review estimated that ~20% of PD patients have cognitive impairment at the time of diagnosis, although their overall older age [mean 71.3 ± 7.5 years, vs. 66.1 ± 12.1 years in our study) is likely to have contributed to the higher rate of cognitive impairment^56^. These observations are in line with studies comparing PARK-LRRK2 vs. idiopathic PD that, importantly, have adjusted for multiple potential confounders, including age, disease duration, and levodopa-equivalent dosages^51,57,58^.

In this work, a total of 283 *LRRK2* variants were documented in patients with different diseases, and 211 were included and classified as pathogenic, likely pathogenic, or VUS according to the ACMG criteria in patients with PD. Looking at the pathogenicity classifications, a challenge became obvious. As can be seen in Fig. 3, the distribution of pathogenicity classifications using a commonly applied tool, i.e., Varsome was quite different from the manually curated one. Varsome often favored the classification of VUS as “likely benign”, which would mean that the patient/variant is not included in MDSGene and not considered for clinical trials. The observed discrepancies underline the need for careful checking of outputs from automated scoring systems by experts in the field^59^. In the manual pathogenicity scoring, all rare variants with a high CADD score were classified as VUS because it cannot be entirely ruled out that they do not affect the development of the disease. However, manual pathogenicity scoring according to ACMG is also challenging, as the criteria for interpretation are subjective and lead to classification inconsistencies^60^. Importantly, the pathogenicity score is only an estimate and based on current knowledge, which may change over time as new insights become available. For example, variants currently classified as VUS may at some point be considered (likely) pathogenic if they are found to segregate in additional families and/or are demonstrated to have relevant functional/biological effects (e.g., elevated LRRK2 kinase activity). Currently, functional evidence is factored into pathogenicity scoring for a few variants only, since for most variants, little research has been done. One notable example is the novel p.F1700L variant in *LRRK2* with functional support of pathogenicity^19^. Therefore, research studies that examine the impact of different *LRRK2* variants on kinase activity on a large scale^18^, are highly important to the field and are currently being generated^61^. Ultimately, understanding the functional impact of specific variants, which, given LRRK2’s wide-ranging physiological roles, may extend beyond the effects on kinase activity, and result in novel therapeutic targets^62,63^.

Notably, an increasing number of publications suggest that *LRRK2* has a broader role beyond its link to classical PD. This includes its potential, albeit probably rare^34^, involvement in the pathogenesis of atypical parkinsonian disorders, since patients harboring potentially pathogenic variants in *LRRK2* have been reported with MSA^27,28,64,65^, PSP^23,29–31,64,66^, CBD^29,32^, and DLB^23^. Of note, most of these patients carry only VUS (8/15). The possible role of *LRRK2* variants in atypical parkinsonian disorders accords with early observations of a broad pathological spectrum in the brains of patients with *LRRK2* variants, involving aggregation of alpha-synuclein, tau, and other proteins^67–69^. Importantly, long-term follow-up of patients initially diagnosed with PD may reveal additional patients with atypical parkinsonian disorders based on the subsequent clinical course or autopsy results, as illustrated by a patient reported as PD^70^, but later found to have MSA-parkinsonian variant (MSA-P)^71^. A further link between *LRRK2* and atypical parkinsonian disorders comes from a recently published genome-wide association study (GWAS) involving 1001 White European-ancestry patients, which suggested a role of common *LRRK2* variation in the survival of PSP patients^72^. Interestingly, *LRRK2* also has a role outside neurological disorders. Notably, *LRRK2* shows its highest expression in blood and lung. It has repeatedly been linked to inflammatory and infectious diseases^73–77^ and postulated to be a link between gut inflammation and PD^63,78^^,^. The observation of several somatic loss-of-function variants in *LRRK2* in breast cancer cells^40^ and germline variants in malignant mesothelioma^79^ are also interesting. However, patients with non-PD phenotypes often carried VUS, making their disease-causing role less clear.

Our study had some limitations that will be addressed when future study and report designs take these shortcomings into account. One of the biggest challenges for this systematic review was the lack of comprehensive reporting of demographic and clinical features. Missing data in up to 99.9% of the patients were observed for some of the extracted variables (Supplementary Table 6). Even the four cardinal clinical features of PD were unreported for 25.7–88.7% of patients (Fig. 5). Furthermore, for instance, the presence or absence of sleep disorder was reported in only 3.7% of cases, making it challenging to conclude whether rapid eye movement sleep behavior disorder (RBD) - increasingly recognized to be a predictor of poorer prognosis in PD^80^ - is less common in PARK-LRRK2 compared to idiopathic PD, as suggested by some authors^51,81^. Non-reporting of features could be linked to the assumption that the authors of the papers with missing data implied the presence of certain symptoms (e.g., the cardinal features) or the absence of symptoms that are only rarely observed, or did not pay attention to a feature (absence of an examination)^3^. A recognized limitation of clinic-based studies is that severely disabled patients may no longer be attending follow-up^82^, or they may be unable to participate in research protocols^83^. In any case, the extent of missing data is an alarming observation, and efforts need to be undertaken to improve this situation, including collecting and reporting longitudinal data on disease progression.

Finally, modifiers of disease penetrance and AAO are areas of significant interest but were beyond the scope of this review; readers are referred to published works that have examined the effects of other genetic (including polygenic)^84,85^, ethno-geographic^86^, and environmental factors^87^, and the interactions thereof^88^. We also did not assess the possible protective effect of *LRRK2* p.G2019S among *GBA1* variant carriers^89–91^, which is an intriguing observation that opens up new questions and avenues for research. It further remains to be seen how the mutational spectrum of *LRRK2* will expand, especially when the whole genome is increasingly being sequenced, and including populations that have hitherto been underrepresented in genetic research^6,92,93^. This approach will furthermore elucidate whether phenotypes or genetic factors in specific communities bias the phenotypic association data. The current emphasis on p.G2019S may be driven, in part, by the fact that many studies have screened specifically for this (and a few other selected) variants.

In conclusion, we performed a systematic literature review, curated and analyzed the data, and present insights from the largest database on PARK-LRRK2 to date. This review can be used to identify pathogenic variants and elucidate their demographic and phenotypic spectrum. Different filter options are available on the MDSGene website (https://www.mdsgene.org/), which also provides published information on in-vivo and in-vitro measurements of LRRK2 kinase activity. This database provides an important resource, especially in light of the emerging molecular-based therapies. However, missing data, especially detailed clinical information, in the publications, and the current limitations in variant interpretation, i.e., the high number of VUS, are important challenges that must be addressed to enable optimal selection and stratification of patients in ongoing and future clinical trials. Thus, this review contributes to improving precision medicine in PARK-LRRK2 patients.

## Methods

### Literature search and data collection process

For the literature search, we used the PubMed database (https://pubmed.ncbi.nlm.nih.gov/). The search term followed the MDSGene´s format (Supplementary Table 11), and articles were screened stepwise based on title, abstract, and full text. We included articles that reported at least one individual with a *LRRK2* variant that was considered potentially disease-causing by the authors. Further, we evaluated the literature cited in the included articles and used the Human Gene Mutation Database (HGMD) professional (https://apps.ingenuity.com/ingsso/login)^94^ to identify additional eligible papers.

Demographic, genetic, and clinical information from all eligible papers were extracted using the MDSGene protocol^4^. Evaluated variables are listed in Supplementary Table 5.

### Inclusion and exclusion criteria for patients and genetic variants

We only included patients with PD and a potentially pathogenic *LRRK2* variant. Unaffected mutation carriers, patients with atypical parkinsonian disorders (dementia with Lewy bodies [DLB], multiple system atrophy [MSA], progressive supranuclear palsy [PSP], and corticobasal degeneration/syndrome [CBD/CBS]), and patients with non-movement disorder conditions were also extracted but excluded from being displayed on the MDSGene database and from the analyses for PD-related genotype-phenotype correlations. Where authors and patient details suggested duplicate reporting of the same individual, we combined information to create a single entry. Patients who carried additional potentially PD-causing variants were also excluded. Although *LRRK2* is inherited in an autosomal dominant manner, patients with heterozygous and homozygous variants were included as before^4^ since previous literature indicated that there is no dosage effect^95^. We excluded *LRRK2* variants that had a minor allele frequency (MAF) of >1% in any ethnicity reported in the gnomAD Browser (https://gnomad.broadinstitute.org/). Carriers of benign or likely benign *LRRK2* variants were also excluded.

### Pathogenicity scoring

All variants were mapped to GRCh37/hg19, and the nomenclature is based on the transcript ENST00000298910 for *LRRK2*. For pathogenicity scoring, we followed the recommendations of the American College of Medical Genetics and Genomics (ACMG)^26^. For this, we used two publicly available online tools, i.e., VarSome (https://varsome.com/) and Franklin (https://franklin.genoox.com/). If there was a discrepancy between the two tools or the score was not plausible, variants were manually scored according to the ACMG recommendations^26^. For this, we applied the CADD score^96^ as an in-silico measurement and data from the functional testing for 100 *LRRK2* variants^18^. Variants categorized as “pathogenic”, “likely pathogenic”, or “variant of uncertain significance” (VUS) were considered as potentially disease-causing variants and included in the MDSGene database and the analyses.

## Supplementary information

Supplement

## Data Availability

All demographic, clinical, and genetic information is available on the MDSGene website in a filterable fashion (https://www.mdsgene.org/).
