# Supplementary material for: Updated MDSGene review on the clinical and genetic spectrum of LRRK2 variants in Parkinson´s disease
Source: NPJ Parkinsons Dis. 2025 Feb 17;11:30. doi: 10.1038/s41531-025-00881-9 (PMC11832785; doi:10.1038/s41531-025-00881-9)
Supplement: Supplementary file 1 — Supplement [file 41531_2025_881_MOESM1_ESM.docx]

**Supporting Information**

***Supplementary Table 1*:** Documented *LRRK2* variants in all extracted variant carriers.

**A:** Variants classified as pathogenic, likely pathogenic, likely benign, or benign

| **Pathogenic** | **Likely Pathogenic** | **Likely Benign** | **Benign** |
| --- | --- | --- | --- |
| R1325Q | R767H | H115P | IVS5+33T>C |
| N1437H | R1067Q | L119P | IVS19-10dupT |
| R1441C | N1221K | IVS4-38A>G | R1514Q |
| R1441G | S1403R | M262V |  |
| R1441H | N1437D | P755L |  |
| R1441S | N1437S | S865F |  |
| A1442P | I1658F | C925Y |  |
| V1447M | R1728H | L1281L |  |
| Y1699C | S1761R | I1371V |  |
| F1700L | S1954F^1^ | R1441R |  |
| L1795F | I2020L | Y1527Y |  |
| G2019S | I2020S | Y2018Y |  |
| I2020T | T2031S | H2391Q |  |

^1^ This variant was only found in a patient with breast cancer and not included in MDSGene

**B:** Variants classified as VUS

| E10K | M579V | H1019R | S1445F | Y1649S | D2175H |
| --- | --- | --- | --- | --- | --- |
| K53R | I610T | Q1029H | P1446L | P1661T | Y2189C |
| M100T | K616R | N1089S | V1450I | R1677S | E2230K |
| Q116R | S633F | S1096C | G1451S | I1691T | H2236R |
| S128G | T646A | L1114L | A1464G | R1693Q | N2251T |
| L153W | A654V | S1120P | K1468E | S1706* | N2261K |
| N173S | S661F | I1122V | P1480L | R1707K | G2294R |
| S192A | I675M | L1128M | A1481T | L1712F | N2308D |
| E193A | F676S | A1151T | R1483Q | R1725Q | T2310M |
| E193K | S722N | C1152F | A1490V | S1752C | N2313S |
| A211V | M724V | S1159R | T1491del | D1756G | I2323F |
| C228S | D734N | L1165P | E1492K | D1756Y | I2336V |
| H230R | K739R | S1181Y | R1501W | H1758P | S2350I |
| N238I | S764N | I1192M | I1505Rfs*16 | R1771T | T2356I |
| Y250C | R767C | I1192V | S1508G | K1772del | V2390A |
| E255K | R772* | A1215T | G1520A | C1774Y | V2390M |
| K258N | T776M | H1216R | V1541M | T1806I | E2395K |
| A259E | I777M | S1228T | I1548V | G1819R | M2408I |
| L286V | S784R | T1271I | R1552* | Q1823K | T2423S |
| V291A | I786F | W1295R | L1583R | M1869T | I2434V |
| A306V | R792K | L1304F | A1589S | M1869V | L2439I |
| L308M | R793M | R1334Q | V1613A | D1873H | A2461V |
| A312V | S827F | I1339M | V1615M | E1874* | L2463P |
| T320S | I844N | V1340M | V1615V | D1887N | Q2490H |
| S231P | M848T | Q1353K | E1616K | G1891A | T2494I |
| N335S | S858* | V1369A | P1619L | L1914I | V2495I |
| A370T | S885C | V1373M | K1620R | R1941H | T2524A |
| L378F | E899D | Q1379R | H1621R | E1948Q | IVS4-68T>C |
| M379I | L922* | L1388I | H1621Q | Q1961R | IVS8+1G>A |
| I388T | Q923H | V1389I | P1622L | I1991V | IVS19+5delGTAA |
| V414I | Q930R | R1398C | P1622P | Y2006H | IVS30+1G>C |
| A419V | D941Y | R1398R | P1622T | A2010T | IVS31+3A>G |
| L425P | D944V | Y1402C | K1623E | I2012T | IVS33+6T>A |
| L437S | R948Q | H1405Q | I1626V | L2063* | IVS33+78T>C |
| S438P | S958L | A1413T | S1627L | V2074I | IVS33+70T>C |
| A459S | D972G | R1320S | S1627T | E2108K | IVS33+79T>C |
| S461I | S973G | E1427G | R1628C | N2133S | IVS36-29G>T |
| N474S | S973N | F1436L | R1639Kfs*13 | C2139S | IVS40-28A>T |
| R521G | R981K | A1440P | Y1645S | V2150I | IVS49+178A>G |
| K535Nfs*13 | S1007T | S1445C | Q1648R | W2168L | Exon 49 deletion |
| K544E |  |  |  |  |  |

***Supplementary Table 2*:** Numbers and frequencies of pathogenic or likely pathogenic *LRRK2* variants in affected PD patients and unaffected individuals as reported in the literature.

| **Variants** | **Number of affected patients (frequency)** | | **Number of unaffected carriers** |
| --- | --- | --- | --- |
| **Pathogenic variants** | | | |
| G2019S | 2480 | (73.22%) | 743 |
| R1441G | 195 | (5.76%) | 90 |
| R1441C | 99 | (2.92%) | 17 |
| I2020T | 45 | (1.33%) | 5 |
| R1441H | 40 | (1.18%) | 4 |
| Y1699C | 33 | (0.97%) | 0 |
| L1795F | 22 | (0.65%) | 3 |
| N1437H | 14 | (0.41%) | 1 |
| V1447M | 6 | (0.18%) | 0 |
| R1441S | 4 | (0.12%) | 0 |
| R1325Q | 3 | (0.09%) | 6 |
| A1442P | 1 | (0.03%) | 0 |
| **Likely pathogenic variants** | | |  |
| R1067Q | 9 | (0.27%) | 0 |
| N1437D | 7 | (0.21%) | 0 |
| S1761R | 7 | (0.21%) | 0 |
| N1437S | 5 | (0.15%) | 0 |
| F1700L | 2 | (0.06%) | 0 |
| I1658F | 2 | (0.06%) | 0 |
| N1221K | 2 | (0.06%) | 0 |
| I2020L | 1 | (0.03%) | 0 |
| I2020S | 1 | (0.03%) | 0 |
| R767H | 1 | (0.03%) | 0 |
| T2031S | 1 | (0.03%) | 0 |
| R1728H | 1 | (0.03%) | 0 |
| S1403R | 1 | (0.03%) | 0 |
| S1954F* | 0 | (0.00%) | 0 |

* found in 1 patient with breast cancer

***Supplementary Table 3***: Characteristics of PD patients with *LRRK2* and additional potentially pathogenic variant(s) in another PD gene.

| **PMID,**  **Author, year** | **family_ID,**  **individual_ID** | **Sex** | **Variant 1** | **Variant 2** | **Variant 3** | **AAO (years)** | **Reported main clinical features** |
| --- | --- | --- | --- | --- | --- | --- | --- |
| 15925109,  Mata, 2005 | PE040,  1i | male | *LRRK2*  c.4321C>G  p.Arg1441Gly | *LRRK2*  c.4937T>C  p.Met1646Thr | *PRKN*  c.574A>G  p.Met192Val | 57 | Bradykinesia, resting tremor, rigidity, good levodopa response |
| 17095157,  Dächsel, 2006 | PJ089,  1i | female | *LRRK2*  c.4321C>G  p.Arg1441Gly | *PRKN*  c.155delA  p.Asn52Metfs*29 | NA | 61 | Bradykinesia, rigidity, good levodopa response |
| 16643318,  Mata, 2006 | i1,  PE055 | male | *LRRK2*  c.6055G>A  p.Gly2019Ser | *PRKN*  c.574A>G  p.Met192Val | NA | 56 | Bradykinesia, resting tremor, rigidity, good levodopa response |
| 17469194,  Ferreira, 2007 | LISB F22,  22_01 | male | *LRRK2*  c.6055G>A  p.Gly2019Ser | *LRRK2*  c.1936A>G  p.Thr646Ala | *PRKN*  c.823C>T  p.Arg275Trp | 42 | Tremor, UPDRS III 17, H&Y 2.5, good levodopa response, non-motor symptoms |
| 17469194,  Ferreira, 2007 | LISB F24,  24_02 | male | *LRRK2*  c.6055G>A  p.Gly2019Ser | *PRKN*  c.155delA  p.Asn52Metfs*29 | NA | 72 | Tremor, UPDRS III 28, H&Y 2, good levodopa response, non-motor symptoms |
| 17388990,  Illarioshkin, 2007 | i1,  PD84 | female | *LRRK2*  c.6055G>A  p.Gly2019Ser | *PRKN*  Exon 5 duplication | NA | 39 | Bradykinesia, resting tremor, rigidity, postural instability, dyskinesia, dystonia |
| 18211709,  Bras, 2008 | i1,  S7 | NA | *LRRK2*  c.6055G>A  p.Gly2019Ser | *PRKN*  Exon 9 duplication | NA | 38 | Motor symptoms (NA) |
| 19473361,  Gao, 2009 | i1,  6 | female | *LRRK2*  c.6055G>A  p.Gly2019Ser | *PRKN*  Exon 4-6 deletion | NA | 30 | Bradykinesia, rigidity, dystonia, good levodopa response, UPDRS III 10, H&Y 2 |
| 20837857,  Alcalay, 2010 | i1,  1i | male | *LRRK2*  c.6055G>A  p.Gly2019Ser | *PRKN*  Exon 5-6 deletion | NA | 41 | Bradykinesia |
| 20837857,  Alcalay, 2010 | i2,  1i | male | *LRRK2*  c.6055G>A  p.Gly2019Ser | *GBA1*  c.1448T>C  p.Leu483Pro | NA | 31 | Bradykinesia,  UPDRS III 24 |
| 20837857,  Alcalay, 2010 | i3,  1i | male | *LRRK2*  c.6055G>A  p.Gly2019Ser | *GBA1*  c.1226A>G  p.Asn409Ser | NA | 47 | Bradykinesia,  UPDRS III 20 |
| 20837857,  Alcalay, 2010 | i4,  1i | female | *LRRK2*  c.6055G>A  p.Gly2019Ser | *GBA1*  c.1226A>G  p.Asn409Ser | NA | 49 | Bradykinesia,  UPDRS III 21 |
| 18644660,  Marras, 2010 | i1,  4539 | NA | *LRRK2*  c.6055G>A  p.Gly2019Ser | *PRKN*  Exon 2 deletion | NA | 43 | Motor symptoms (NA), UPDRS ADL score of 9/56, UPDRS motor score of 35/108 |
| 18644660,  Marras, 2010 | i1,  4612 | NA | *LRRK2*  c.6055G>A  p.Gly2019Ser | *PRKN*  Exon 2 deletion | NA | 68 | Motor symptoms (NA), UPDRS ADL score of 21/56,  UPDRS motor score of 41/108 |
| 23124679,  Itokawa, 2013 | i1,  1i | male | *LRRK2*  c.7153G>A  p.Gly2385Arg | *LRRK2*  c.4348G>A  p.Val1450Ile | *SNCA*  Exon 1-6 duplication | 20 | Bradykinesia, resting tremor, rigidity, dystonia, motor fluctuations, psychotic symptoms |
| 23664753,  Ling, 2013 | i1,  1i | female | *LRRK2*  c.6055G>A  p.Gly2019Ser | *MAPT*  c.370C>G  p.Gln124Glu | NA | 73 | Bradykinesia, tremor, rigidity, good levodopa response |
| 24816003,  Cilia, 2014 | i1,  1i | male | *LRRK2*  c.4321C>T  p.Arg1441Cys | *GBA1*  c.1226A>G  p.Asn409Ser | NA | 44 | Bradykinesia, good levodopa response, dyskinesia, motor fluctuations, non-motor symptoms |
| 24816003,  Cilia, 2014 | i2,  1i | male | *LRRK2*  c.6055G>A  p.Gly2019Ser | *GBA1*  c.1226A>G  p.Asn409Ser | NA | 49 | Resting tremor, UPDRS II 14, good levodopa response, dyskinesia, motor fluctuations, psychotic symptoms |
| 25899316,  Jankovic, 2015 | i1,  2 | female | *LRRK2*  c.4522A>G  p.Ser1508Gly | *GBA1*  c.1226A>G  p.Asn409Ser | NA | 58 | Bradykinesia, rigidity |
| 25952961,  Spitz, 2015 | i1,  III-6 | female | *LRRK2*  c.6055G>A  p.Gly2019Ser | *GBA1*  c.1448T>C  p.Leu483Pro | NA | 38 | Rigiidity, good levodopa response, UPDRS III 26, non-motor symptoms |
| 30235682,  Bartoníková, 2018 | i1,  5 | female | *LRRK2*  c.437-68T>C  IVS4-68T>C | *SNCA*  c.*77C>T | NA | 60 | Resting tremor |
| 30528841,  Blauwendraat, 2019 | i1,  JHND1206 | female | *LRRK2*  c.6055G>A  p.Gly2019Ser | *GBA1*  c.1226A>G  p.Asn409Ser | NA | NA | Motor symptoms (NA) |
| 31324919,  Tan, 2019 | i1,  1i | NA | *LRRK2*  c.6055G>A  p.Gly2019Ser | *GBA1*  p.Glu326Lys/ p.Pro122His | NA | NA | Bradykinesia |
| 32866938,  Mangone, 2020 | i1,  1i | female | *LRRK2*  c.6055G>A  p.Gly2019Ser | *GBA1*  c.1448T>C  p.Leu483Pro | NA | 32 | Motor symptoms (NA), UPDRS III 18, H&Y 4 |
| 32866938,  Mangone, 2020 | i2,  1i | male | *LRRK2*  c.6055G>A  p.Gly2019Ser | *GBA1*  c.247C>T  p.Arg83Cys | NA | 44 | Motor symptoms (NA), UPDRS III 23, H&Y 3 |
| 32794657,  Zheng, 2020 | i1,  1i | NA | *LRRK2*  c.1256C>T  p.Ala419Val | *CHCHD2*  c.182C>T  p.Thr61Ile | NA | NA | Motor symptoms (NA) |
| 33836114,  Bright, 2021 | i1,  ESB-01-02 | male | *LRRK2*  c.6055G>A  p.Gly2019Ser | *GBA1*  either p.Asn409Ser or a stop codon | NA | NA | Motor symptoms (NA) |
| 34076298,  Isonaka, 2021 | i1,  1i | female | *LRRK2*  c.6055G>A  p.Gly2019Ser | *GBA1*  c.1226A>G  p.Asn409Ser | NA | 58 | Motor symptoms (NA) |
| 34076298,  Isonaka, 2021 | i2,  1i | male | *LRRK2*  c.6055G>A  p.Gly2019Ser | *GBA1*  c.1226A>G  p.Asn409Ser | NA | 66 | Motor symptoms (NA) |
| 33742109,  Kanaya, 2021 | i1,  57 | NA | *LRRK2*  c.713A>T  p.Asn238Ile | *PINK1*  Exon 1 deletion | NA | 45 | Bradykinesia |
| 32557143,  Smaili, 2021 | i1,  3848 | male | *LRRK2*  c.4136A>G  Gln1379Arg | *PINK1*  c.851C>T  p.Ser284Phe | NA | 70 | Bradykinesia, resting tremor, postural instability, dyskinesia, motor fluctuations, UPDRS III 66, H&Y 4, non-motor symptoms |
| 35466950,  Lee, 2022 | i1,  1i | NA | *LRRK2*  c.6055G>A  p.Gly2019Ser | *GBA1*  c.1226A>G  p.Asn409Ser | NA | NA | Bradykinesia |
| 36879366,  Do, 2023 | i1,  PD149 | female | *LRRK2*  Exon 49 deletion | *PRKN*  Exon 4 deletion | *MAPT* variant unclear | 41 | Bradykinesia |
| 38173558,  Landoulsi, 2023 | i1,  1i | NA | *LRRK2*  c.7224G>A  p.Met2408Ile | *PRKN*  c.766C>T  p.Arg256Cys | NA | NA | Bradykinesia |
| 37256495,  Smaili, 2023 | i1,  3851 | male | *LRRK2*  c.2025A>G  p.Ile675Met | *MAPT*  c.1280C>T  p.Ser427Phe | NA | 37 | Bradykinesia |
| 37256495,  Smaili, 2023 | i2,  3909 | female | *LRRK2*  c.6220G>A  p.Val2074Ile | *DNAJC6*  c.1723C>G  p.Pro575Ala | *DNAJC13*  c.1268C>T  p.Ala423Val | 60 | Bradykinesia |
| 37256495,  Smaili, 2023 | i3,  3940 | male | *LRRK2*  c.6566A>G  p.Tyr2189Cys | *DJ-1*  c.328A>C  p.Thr110Pro | *DJ-1*  c.423C>G  p.Tyr141* | 27 | Bradykinesia |
| 37256495,  Smaili, 2023 | i4,  3947 | male | *LRRK2*  c.6566A>G  p.Tyr2189Cys | *VPS13C*  c.1880T>C  p.Val627Ala | *MAPT*  c.1280C>T  p.Ser427Phe | 72 | Bradykinesia |
| 37256495,  Smaili, 2023 | i5,  3316 | male | *LRRK2*  c.2915A>G  p.Asp972Gly | *DNAJC6*  c.1493G>C  p.Cys498Ser | *GBA1*  c.1504C>T  p.Arg502Cys | 48 | Bradykinesia |
| 37201327,  Sun, 2023 | i1,  III-5 | female | *LRRK2*  c.4309A>G  p.Asn1437Asp | *VPS35*  c.151G>A  p.Gly51Ser | NA | 48 | Bradykinesia, resting tremor, rigidity, postural instability, motor fluctuations, UPDRS III 33, H&Y 3, depression |
| 37201327,  Sun, 2023 | i1,  III-3 | male | *LRRK2*  c.4309A>G  p.Asn1437Asp | *VPS35*  c.151G>A  p.Gly51Ser | NA | 55 | Bradykinesia resting tremor, rigidity, postural instability, motor fluctuations, UPDRS III 56, H&Y 3, non-motor symptoms |
| 37301871,  Tang, 2023 | i1,  1i | male | *LRRK2*  c.691T>C  p.Ser231Pro | *GBA1*  c.1448T>C  p.Leu483Pro | NA | NA | Bradykinesia, rigidity, dystonia, good levodopa response, UPDRS III (OFF/ON) 37/15, non-motor symptoms |
| 38901781,  Tezuka, 2024 | Family A,  A1 | female | *LRRK2*  c.3358T>C  p.Ser1120Pro | *GBA1*  c.1448T>C  p.Leu483Pro | NA | 40 | Bradykinesia, resting tremor, rigidity, postural instability, dyskinesia, motor fluctuations, autonomic symptoms |
| 38702337,  Tropea, 2024 | i1,  1i | NA | *LRRK2*  c.6055G>A  p.Gly2019Ser | *GBA1*  c.1226A>G  p.Asn409Ser | NA | NA | Motor symptoms (NA) |
| 38702337,  Tropea, 2024 | i2,  1i | NA | *LRRK2*  c.6055G>A  p.Gly2019Ser | *GBA1*  c.1226A>G  p.Asn409Ser | NA | NA | Motor symptoms (NA) |
| 38702337,  Tropea, 2024 | i3,  1i | NA | *LRRK2*  c.6055G>A  p.Gly2019Ser | *GBA1*  c.1226A>G  p.Asn409Ser | NA | NA | Motor symptoms (NA) |

UPDRS - Unified Parkinson’s disease Rating Scale; H&Y – Hoehn and Yahr stage; NA – not available

***Supplementary Table 4*:** Age at onset (AAO) of PD in the included patients with pathogenic or likely pathogenic variants, in all included patients with VUS, and in all included patients with available information, respectively. The first number indicates the number of patients, and the number in parentheses is the respective frequency.

| **Age at onset** | **Patients with (likely) pathogenic variants** | | **Patients with VUS** | | **All patients** | |
| --- | --- | --- | --- | --- | --- | --- |
| 0-9 | 0 | (0.0%) | 0 | (0.0%) | 0 | (0.0%) |
| 10-19 | 0 | (0.0%) | 0 | (0.0%) | 0 | (0.0%) |
| 20-29 | 13 | (1.5%) | 4 | (2.8%) | 17 | (1.6%) |
| 30-39 | 64 | (7.2%) | 15 | (10.3%) | 79 | (7.6%) |
| 40-49 | 201 | (22.5%) | 45 | (31.0%) | 246 | (23.7%) |
| 50-59 | 267 | (29.9%) | 24 | (16.6%) | 291 | (28.0%) |
| 60-69 | 224 | (25.1%) | 34 | (23.4%) | 258 | (24.8%) |
| 70-79 | 109 | (12.2%) | 23 | (15.9%) | 132 | (12.7%) |
| 80-89 | 15 | (1.7%) | 0 | (0.0%) | 15 | (1.4%) |
| 90-99 | 1 | (0.1%) | 0 | (0.0%) | 1 | (0.1%) |
| known | 894 | (100.0%) | 145 | (100.0%) | 1039 | (100.0%) |
| unknown | 2087 |  | 261 |  | 2348 |  |
| total | 2981 |  | 406 |  | 3387 |  |

***Supplementary Table 5:*** List of variables. Demographic, clinical, and genetic information extracted using the MDSGene protocol.

**Patient characterization**

individual patient ID

family ID

**Demographic information**

ethnicity

country of origin

sex

**Genetic information**

genetic status of all tested family members with number of homozygous, heterozygous and wildtype ones in affected and unaffected state

human genome version number

genome build or accession ID

gene to which the variant refers

physical location of the variant (on the plus strand)

observed mutated bases (on the plus strand)

reference bases (on the plus strand)

genomic description of the variant according to the nomenclature of the human genome variation society (HGVS)

coding description of the variant according to the nomenclature of the HGVS

protein description of the variant according to the nomenclature of the HGVS

genotype of the respective variant

type of variant

highest minor allele frequency (MAF) of the respective variant found in gnomAD

pathogenicity status classified as likely benign, benign, VUS (variant of unknown significance), likely pathogenic or pathogenic

CADD score as one parameter for pathogenicity scoring

Functional evidence

**Clinical information**

general

positive family history for the variant associated phenotype

age at examination in years

age at disease onset in years

disease duration in years

age at clinical diagnosis in years

age at death

presence of parkinsonian syndrome

motor instrument like UPDRS III or Hoehn&Yahr and, if available, their scoring

presence of non-motor signs and symptoms associated with a parkinsonian syndrome

non-motor symptoms instrument

presence of atypical signs and symptoms for a parkinsonian syndrome

reported initial signs and symptoms

motor signs and symptoms

bradykinesia

tremor (in general: HP:0001337)

rest tremor

action tremor

postural tremor

dystonic tremor

rigidity

postural instability

levodopa responsiveness

levodopa response quantification classified as minimal/intermittent, moderate, or good/excellent

dyskinesia

dystonia

hyperreflexia

diurnal fluctuations as motor symptom fluctuations throughout the day

sleep benefit as restoration of mobility upon awakening from sleep

motor fluctuations in context of medication-intake

non-motor signs and symptoms

hyposmia

depression

depression scale

anxiety

anxiety scale

psychotic symptoms

psychosis scale

sleep disorder

cognitive decline

autonomic symptoms

***Supplementary Table 6*:** Information on reported signs and symptoms in the patients with pathogenic or likely pathogenic variants (n=2,981). The first number indicates the number of patients, and the number in parentheses is the respective frequency.

| **Symptoms** | **Present** | | **Absent** | | **Unknown** | |
| --- | --- | --- | --- | --- | --- | --- |
| Motor symptoms | 2981 | (100.0%) | 0 | (0.0%) | 0 | (0.0%) |
| Parkinsonism | 2981 | (100.0%) | 0 | (0.0%) | 0 | (0.0%) |
| Bradykinesia | 2208 | (74.1%) | 8 | (0.3%) | 765 | (25.7%) |
| Rigidity | 642 | (21.5%) | 9 | (0.3%) | 2330 | (78.2%) |
| Tremor (any or unspecified) | 572 | (19.2%) | 63 | (2.1%) | 2346 | (78.7%) |
| Resting tremor | 366 | (12.3%) | 69 | (2.3%) | 2546 | (85.4%) |
| Action tremor | 26 | (0.9%) | 10 | (0.3%) | 2945 | (98.8%) |
| Postural tremor | 18 | (0.6%) | 9 | (0.3%) | 2954 | (99.1%) |
| Dystonic tremor | 0 | (0.0%) | 3 | (0.1%) | 2978 | (99.9%) |
| Postural instability | 246 | (8.3%) | 90 | (3.0%) | 2645 | (88.7%) |
| Dyskinesia | 206 | (6.9%) | 92 | (3.1%) | 2683 | (90.0%) |
| Dystonia | 66 | (2.2%) | 100 | (3.4%) | 2815 | (94.4%) |
| Hyperreflexia | 13 | (0.4%) | 50 | (1.7%) | 2918 | (97.9%) |
| Diurnal fluctuations | 2 | (0.1%) | 0 | (0.0%) | 2979 | (99.9%) |
| Sleep benefit | 18 | (0.6%) | 61 | (2.0%) | 2902 | (97.3%) |
| Motor fluctuations | 147 | (4.9%) | 71 | (2.4%) | 2763 | (92.7%) |
| Non-motor symtpoms | 278 | (9.3%) | 28 | (0.9%) | 2675 | (89.7%) |
| Olfaction symptoms | 38 | (1.3%) | 10 | (0.3%) | 2933 | (98.4%) |
| Depression | 109 | (3.7%) | 157 | (5.3%) | 2715 | (91.1%) |
| Anxiety | 49 | (1.6%) | 114 | (3.8%) | 2818 | (94.5%) |
| Psychotic symptoms | 65 | (2.2%) | 140 | (4.7%) | 2776 | (93.1%) |
| Sleep disorder | 61 | (2.0%) | 52 | (1.7%) | 2868 | (96.2%) |
| Cognitive decline | 110 | (3.7%) | 323 | (10.8%) | 2548 | (85.5%) |
| Autonomic symptoms | 59 | (2.0%) | 84 | (2.8%) | 2838 | (95.2%) |
| Atypical parkinsonism symptoms | 8 | (0.3%) | 316 | (10.6%) | 2657 | (89.1%) |

***Supplementary Table 7*:** Information on reported signs and symptoms in the patients with VUS (n=406). The first number indicates the number of patients, and the number in parentheses is the respective frequency.

| **Symptoms** | **Present** | | **Absent** | | **Unknown** | |
| --- | --- | --- | --- | --- | --- | --- |
| Motor symptoms | 406 | (100.0%) | 0 | (0.0%) | 0 | (0.0%) |
| Parkinsonism | 406 | (100.0%) | 0 | (0.0%) | 0 | (0.0%) |
| Bradykinesia | 279 | (68.7%) | 2 | (0.5%) | 125 | (30.8%) |
| Rigidity | 73 | (18.0%) | 3 | (0.7%) | 330 | (81.3%) |
| Tremor (any or unspecified) | 81 | (20.0%) | 7 | (1.7%) | 318 | (78.3%) |
| Resting tremor | 58 | (14.3%) | 16 | (3.9%) | 332 | (81.8%) |
| Action tremor | 3 | (0.7%) | 0 | (0.0%) | 403 | (99.3%) |
| Postural tremor | 6 | (1.5%) | 1 | (0.2%) | 399 | (98.3%) |
| Dystonic tremor | 0 | (0.0%) | 0 | (0.0%) | 406 | (100.0%) |
| Postural instability | 30 | (7.4%) | 21 | (5.2%) | 355 | (87.4%) |
| Dyskinesia | 12 | (3.0%) | 17 | (4.2%) | 377 | (92.9%) |
| Dystonia | 5 | (1.2%) | 17 | (4.2%) | 384 | (94.6%) |
| Hyperreflexia | 0 | (0.0%) | 5 | (1.2%) | 401 | (98.8%) |
| Diurnal fluctuations | 0 | (0.0%) | 0 | (0.0%) | 406 | (100.0%) |
| Sleep benefit | 2 | (0.5%) | 2 | (0.5%) | 402 | (99.0%) |
| Motor fluctuations | 11 | (2.7%) | 4 | (1.0%) | 391 | (96.3%) |
| Non-motor symtpoms | 45 | (11.1%) | 0 | (0.0%) | 361 | (88.9%) |
| Olfaction symptoms | 11 | (2.7%) | 4 | (1.0%) | 391 | (96.3%) |
| Depression | 11 | (2.7%) | 11 | (2.7%) | 384 | (94.6%) |
| Anxiety | 2 | (0.5%) | 0 | (0.0%) | 404 | (99.5%) |
| Psychotic symptoms | 5 | (1.2%) | 9 | (2.2%) | 392 | (96.6%) |
| Sleep disorder | 13 | (3.2%) | 8 | (2.0%) | 385 | (94.8%) |
| Cognitive decline | 15 | (3.7%) | 22 | (5.4%) | 369 | (90.9%) |
| Autonomic symptoms | 14 | (3.4%) | 5 | (1.2%) | 387 | (95.3%) |
| Atypical parkinsonism symptoms | 2 | (0.5%) | 6 | (1.5%) | 398 | (98.0%) |

***Supplementary Table 8***: Information on reported signs and symptoms in all included patients (n=3,387). First number indicates the number of patients, number in parentheses is the respective frequency.

| **Symptoms** | **Present** | | **Absent** | | **Unknown** | |
| --- | --- | --- | --- | --- | --- | --- |
| Motor symptoms | 3387 | (100.0%) | 0 | (0.0%) | 0 | (0.0%) |
| Parkinsonism | 3387 | (100.0%) | 0 | (0.0%) | 0 | (0.0%) |
| Bradykinesia | 2487 | (73.4%) | 10 | (0.3%) | 890 | (26.3%) |
| Rigidity | 715 | (21.1%) | 12 | (0.4%) | 2660 | (78.5%) |
| Tremor (any or unspecified) | 653 | (19.3%) | 70 | (2.1%) | 2664 | (78.7%) |
| Resting tremor | 424 | (12.5%) | 85 | (2.5%) | 2878 | (85.0%) |
| Action tremor | 29 | (0.9%) | 10 | (0.3%) | 3348 | (98.8%) |
| Postural tremor | 24 | (0.7%) | 10 | (0.3%) | 3353 | (99.0%) |
| Dystonic tremor | 0 | (0.0%) | 3 | (0.1%) | 3384 | (99.9%) |
| Postural instability | 276 | (8.1%) | 111 | (3.3%) | 3000 | (88.6%) |
| Dyskinesia | 218 | (6.4%) | 109 | (3.2%) | 3060 | (90.3%) |
| Dystonia | 71 | (2.1%) | 117 | (3.5%) | 3199 | (94.4%) |
| Hyperreflexia | 13 | (0.4%) | 55 | (1.6%) | 3319 | (98.0%) |
| Diurnal fluctuations | 2 | (0.1%) | 0 | (0.0%) | 3385 | (99.9%) |
| Sleep benefit | 20 | (0.6%) | 63 | (1.9%) | 3304 | (97.5%) |
| Motor fluctuations | 158 | (4.7%) | 75 | (2.2%) | 3154 | (93.1%) |
| Non-motor symptoms | 323 | (9.5%) | 28 | (0.8%) | 3036 | (89.6%) |
| Olfaction symptoms | 49 | (1.4%) | 14 | (0.4%) | 3324 | (98.1%) |
| Depression | 120 | (3.5%) | 168 | (5.0%) | 3099 | (91.5%) |
| Anxiety | 51 | (1.5%) | 114 | (3.4%) | 3222 | (95.1%) |
| Psychotic symptoms | 70 | (2.1%) | 149 | (4.4%) | 3168 | (93.5%) |
| Sleep disorder | 74 | (2.2%) | 60 | (1.8%) | 3253 | (96.0%) |
| Cognitive decline | 125 | (3.7%) | 345 | (10.2%) | 2917 | (86.1%) |
| Autonomic symptoms | 73 | (2.2%) | 89 | (2.6%) | 3225 | (95.2%) |
| Atypical parkinsonism symptoms | 10 | (0.3%) | 322 | (9.5%) | 3055 | (90.2%) |

***Supplementary Table 9*:** Reported initial signs and symptoms in patients with pathogenic or likely pathogenic variants, in the included patients with VUS, and in all included patients. The first number indicates the number of patients; numbers in parentheses are the respective frequencies among all patients in the same group (middle panel) and patients in the same group with information (right panel).

| **Initial symptoms** | **Patients with (likely) pathogenic variants** | | | **Patients with VUS** | | | **All included patients** | | |
| --- | --- | --- | --- | --- | --- | --- | --- | --- | --- |
| Tremor (any or unspecified) | 240 | (8.1%) | (52.5%) | 19 | (4.7%) | (42.2%) | 259 | (7.6%) | (51.6%) |
| Bradykinesia | 175 | (5.9%) | (38.3%) | 17 | (4.2%) | (37.8%) | 192 | (5.7%) | (38.2%) |
| Rigidity | 77 | (2.6%) | (16.8%) | 5 | (1.2%) | (11.1%) | 82 | (2.4%) | (16.3%) |
| Resting tremor | 61 | (2.0%) | (12.9%) | 11 | (2.7%) | (24.4%) | 72 | (2.1%) | (14.3%) |
| Postural instability | 5 | (0.2%) | (1.1%) | 1 | (0.2%) | (2.2%) | 6 | (0.2%) | (1.2%) |
| Dystonia | 8 | (0.3%) | (1.8%) | 0 | (0.0%) | (0.0%) | 8 | (0.2%) | (1.6%) |
| Anxiety | 3 | (0.1%) | (0.7%) | 0 | (0.0%) | (0.0%) | 3 | (0.1%) | (0.6%) |
| Cognitive decline | 2 | (0.1%) | (0.4%) | 1 | (0.2%) | (2.2%) | 3 | (0.1%) | (0.6%) |
| Sleep disorder | 1 | (0.0%) | (0.2%) | 0 | (0.0%) | (0.0%) | 1 | (0.0%) | (0.2%) |
| Depression | 7 | (0.2%) | (1.5%) | 0 | (0.0%) | (0.0%) | 7 | (0.2%) | (1.4%) |
| Tremor postural | 1 | (0.0%) | (0.2%) | 0 | (0.0%) | (0.0%) | 1 | (0.0%) | (0.2%) |
| Psychotic symptoms | 1 | (0.0%) | (0.2%) | 0 | (0.0%) | (0.0%) | 1 | (0.0%) | (0.2%) |
| Known | 457 | (15.3%) | (100.0%) | 45 | (11.1%) | (100.0%) | 502 | (14.8%) | (100.0%) |
| Unknown | 2524 | (84.7%) |  | 361 | (88.9%) |  | 2885 | (85.2%) |  |
| All | 2981 | (100.0%) |  | 406 | (100.0%) |  | 3387 | (100.0%) |  |

***Supplementary Table 10*:** Levodopa response quantifications in the included PD patients with (likely) pathogenic *LRRK2* variants, in the included PD patients with VUS, and in all included PD patients.

| **Levodopa response** | **Patients with (likely) pathogenic variants** | | **Patients with VUS** | | **All included patients** | |
| --- | --- | --- | --- | --- | --- | --- |
| good/excellent | 344 | (11.5%) | 53 | (13.1%) | 397 | (11.7%) |
| moderate | 5 | (0.2%) | 0 | (0.0%) | 5 | (0.1%) |
| minimal/intermittent | 12 | (0.4%) | 2 | (0.5%) | 14 | (0.4%) |
| yes, undefined | 416 | (14.0%) | 36 | (8.9%) | 452 | (13.3%) |
| no | 2 | (0.1%) | 1 | (0.2%) | 3 | (0.1%) |
| not treated | 18 | (0.6%) | 5 | (1.2%) | 23 | (0.7%) |
| unknown | 2184 | (73.3%) | 309 | (76.1%) | 2493 | (73.6%) |
| total | 2981 | (100.0%) | 406 | 100.0%) | 3387 | (100.0%) |

***Supplementary Table 11:*** Search term for the literature search for *LRRK2* in PubMed

| *LRRK2* | (ataxia OR ataxic OR cerebellar OR channelopathy OR dystonia OR dystonic OR parkinson* OR paroxysmal movement OR tremor OR myoclon* OR chorea OR choreo* OR choreatic OR spastic paraplegia OR spastic paraparesis OR HSP OR Strümpell* OR hyperkinetic OR “movement disorder” OR dyskinesia OR dyskinetic) AND (LRRK2 OR PARK8 OR DARDARIN OR RIPK7 OR ROCO2 OR AURA17 OR DKFZp434H2111 OR FLJ45829 OR RIPK7 OR “Leucin-rich repeat kinase 2” OR 12q12) AND ("english"[Language]) |
| --- | --- |

**A**

28

34

48

86

153

228

397

505

Ethnicity of the included patients with (likely) pathogenic variants Number of patients with missing data: 1502 (50.4%)

Number of patients

|  | p.G2019S | p.I2020T | other variants |
| --- | --- | --- | --- |
| Caucasian | 336 | 0 | 169 |
| Arab | 397 | 0 | 0 |
| Mixed/other | 124 | 0 | 104 |
| Asian | 0 | 25 | 61 |
| Jewish (Ashkenazi) | 153 | 0 | 0 |
| African ancestry | 48 | 0 | 0 |
| Hispanic | 30 | 0 | 4 |
| Jewish (non-Ashkenazi/undefined) | 28 | 0 | 0 |

**B**

28

35

49

153

206

245

397

559

Ethnicity of all included patients Number of patients with missing data: 1715 (50.6%)

Number of patients

|  | p.G2019S | p.A419V | other variants |
| --- | --- | --- | --- |
| Caucasian | 336 | 0 | 223 |
| Arab | 397 | 0 | 0 |
| Mixed/other | 124 | 0 | 121 |
| Asian | 0 | 65 | 141 |
| Jewish (Ashkenazi) | 153 | 0 | 0 |
| African ancestry | 48 | 0 | 1 |
| Hispanic | 30 | 0 | 5 |
| Jewish (non-Ashkenazi/undefined) | 28 | 0 | 0 |

**C**

**D**

***Supplementary Figure 1*:** Origin of included PD patients. **A)** Ethnicities of the included patients carrying (likely) pathogenic variants, with the most frequent variant per ethnicity highlighted. **B)** Ethnicities of all the included patients with the most frequent variant per ethnicity highlighted. **C)** World map for included PD patients with (likely) pathogenic *LRRK2* variants. The country of origin with the highest number of affected *LRRK2* carriers is Tunisia (n = 545, followed by Spain (n = 310) and Italy (n = 133). D**)** World map for all the included PD patients with *LRRK2* variants. The country of origin with the highest number of affected *LRRK2* carriers is Tunisia (n = 553, followed by Spain (n = 313) and Italy (n = 145).

Reported signs and symptoms

|  | (Likely) Pathogenic | VUS | Total |
| --- | --- | --- | --- |
| Motor symptoms | 100% | 100% | 100% |
| Parkinsonism | 100% | 100% | 100% |
| Bradykinesia | 99.639% | 99.2883% | 99.5995% |
| Rigidity | 98.6175% | 96.0526% | 98.3494% |
| Tremor (any or unspecified) | 90.0787% | 92.0455% | 90.3181% |
| Resting tremor | 84.1379% | 78.3784% | 83.3006% |
| Action tremor | 72.2222% | 100% | 74.359% |
| Postural tremor | 66.6667% | 85.7143% | 70.5882% |
| Postural instability | 73.2143% | 58.8235% | 71.3178% |
| Dyskinesia | 69.1275% | 41.3793% | 66.6667% |
| Dystonia | 39.759% | 22.7273% | 37.766% |
| Hyperreflexia | 20.6349% | 0% | 19.1176% |
| Diurnal fluctuations | 100% | 0% | 100% |
| Sleep benefit | 22.7848% | 50% | 24.0964% |
| Motor fluctutations | 67.4312% | 73.3333% | 67.8112% |
| Non-motor symptoms | 90.8497% | 100% | 92.0228% |
| Hyposmia | 79.1667% | 73.3333% | 77.7778% |
| Depression | 40.9774% | 50% | 41.6667% |
| Anxiety | 30.0613% | 100% | 30.9091% |
| Psychotic symptoms | 31.7073% | 35.7143% | 31.9635% |
| Sleep disorder | 53.9823% | 61.9048% | 55.2239% |
| Cognitive decline | 25.4042% | 40.5405% | 26.5957% |
| Autonomic symptoms | 41.2587% | 73.6842% | 45.0617% |
| Atypical parkinsonism symptoms | 2.46914% | 25% | 3.01205% |

***Supplementary Figure 2*:** Percentages of features reported to be present among all patients in the respective group. All reported signs and symptoms (“present”) in the included patients with (likely) pathogenic variants (n=2,981), with VUS (n=406), and in all included PD patients (n=3,387). Of note, there is bias in this presentation since the diagram includes only information from patients with available data (respective feature reported as being present). There may be overreporting of unusual features combined with underreporting of the absence of respective features (missing data), leading to a too-high frequency, especially when the sample size (patients with information) is small.

Initial signs and symptoms

|  | (Likely) Pathogenic | VUS | All |
| --- | --- | --- | --- |
| Tremor (any or unspecified) | 52.5164% | 42.2222% | 51.5936% |
| Bradykinesia | 38.2932% | 37.7778% | 38.247% |
| Rigidity | 16.849% | 11.1111% | 16.3347% |
| Resting tremor | 13.3479% | 24.4444% | 14.3426% |
| Postural instability | 1.09409% | 2.22222% | 1.19522% |
| Dystonia | 1.75055% | 0% | 1.59363% |
| Anxiety | 0.656455% | 0% | 0.59761% |
| Cognitive decline | 0.437637% | 2.22222% | 0.59761% |
| Sleep disorder | 0.218818% | 0% | 0.199203% |
| Depression | 1.53173% | 0% | 1.39442% |
| Postural tremor | 0.218818% | 0% | 0.199203% |
| Psychotic symptoms | 0.218818% | 0% | 0.199203% |

***Supplementary Figure 3*:** Initial signs and symptoms (“present”) in the included patients with (likely) pathogenic variants (red, n=457), with VUS (grey, n=45), and in all included patients with information (blue, n=502), respectively.

Levodopa response

Number of patients

|  | (Likely) Pathogenic | VUS | All |
| --- | --- | --- | --- |
| good/excellent | 344 | 53 | 397 |
| moderate | 5 | 0 | 5 |
| minimal/intermittent | 12 | 2 | 14 |
| yes, undefined | 416 | 36 | 452 |
| no | 2 | 1 | 3 |
| not treated | 18 | 5 | 23 |

***Supplementary Figure 4*:** Levodopa response quantifications in the included PD patients with (likely) pathogenic *LRRK2* variants (red, n=797), in the included PD patients with VUS (grey, n=97), and in all included PD patients with information (blue, n=894), respectively.
